# Descriptive analysis of Thoroughbred horses born in Victoria, Australia, in 2010; barriers to entering training and outcomes on exiting training and racing

**DOI:** 10.1371/journal.pone.0241273

**Published:** 2020-10-28

**Authors:** Meredith L. Flash, Michelle Renwick, James R. Gilkerson, Mark A. Stevenson

**Affiliations:** Asia-Pacific Centre for Animal Health, Faculty of Veterinary and Agricultural Sciences, The University of Melbourne, Parkville, Victoria, Australia; University of Lincoln, UNITED KINGDOM

## Abstract

The reasons for Thoroughbred (TB) horses not entering training or exiting the racing industry, are of interest to regulators, welfare groups and the broader community. Speculation about the outcomes of these horses threatens the community acceptance, or social license, of the TB breeding and racing industries. A representative survey of the 2010 Victorian born TB foal crop was used to determine the outcomes and reasons for exit for horses that had not entered training, or had exited training and racing by eight years of age. Horses exported for racing or breeding (4%), or that were still actively racing (7%) at the start of the follow up period were excluded from the study. An online questionnaire was sent to breeders or trainers of 3,176 TB horses eligible for enrolment in the study. Of the 2,005 (63%) responses received, the two most frequent outcomes were that the horse had either been retired or rehomed (65%), or deceased (16%). For the 1,637 TB horses that had entered training, the majority of retirements were voluntary (59%), followed by involuntary retirements due to health disorders (28%). For TBs that did not have an industry record of entering training (*n* = 368), death (34%), or retirement or being rehomed (27%), were the most frequent barriers to entering training. The median age of retirement for TBs that raced was five (Q1 4; Q3 6) years regardless of sex, or whether their first race start was at two, three or four years of age. Relatively large numbers of horses voluntarily retiring at five-years of age suggests that industry-level, rather than individual horse-level factors are the predominant influences on racing career duration.

## Introduction

The Australian Thoroughbred (TB) industry is an important component of the Victorian (VIC) and broader Australian economies, contributing more than $1.5 billion Australian dollars (AUD) to the VIC economy alone [1, 2]. However, over the last decade, community concern about the outcomes for horses once they have left the racing industry has also developed into concern regarding horse welfare in general, and the number of horses that are bred for racing [3–8]. This has led to an interest in the outcomes for horses that either do not enter training, or that leave the racing industry but do not go back into the TB breeding industry. These concerns have the potential to threaten the continued community approval of the TB racing industry, or their social license to operate [9]. Social license to operate originated in the mining sector and is the recognition of the community as a stakeholder, whose approval an industry requires, to continue to operate [9]. The conditions that community stakeholders require to maintain this approval may go beyond the current regulations and laws under which an industry operates [9]. Over the last 20 years, the number of live foals produced in the Australian TB industry has reduced by nearly a third [10, 11]. Despite the reduced number of horses being bred, there is still a perception that there are too many TB horses and a growing uncertainty about the number that eventually race [3, 8, 12, 13]. A recent United Kingdom (UK) study showed that 6% of foals died within the first two years of life [14]. However, it is difficult to make direct comparisons between the Australian and UK TB cohorts at two years of age, as there are many UK TB horses that are retained for National Hunt races, which do not occur in Australia [14]. Previous Australian research reported that 7% of the foals in a study cohort died before weaning and a further 23% failed to race [15]. Research using cohorts from Australian yearling sales reported that 13–15% of these horses failed to race [15, 16]. These figures, however, are based on small subsets of the Australian horse population and while these earlier reports provide useful estimates of the proportion of horses that fail to race, there is a paucity of population level data available on the reasons that horses did not transition from the stud farm to the racetrack. A recent Australian study of foals born in 2005 and 2010 concluded that subsets of the population potentially overestimated the proportion of TB horses that enter training and racing [17].

Once horses retire from racing, there is little available information about their outcomes, particularly if they leave the TB breeding and racing industries. This has led to conjecture and concern in the broader community, over the outcomes and ongoing welfare of horses once they finish their racing career. Previous Australian research that investigated horses in residence in a racing stable, found that 40% temporarily or permanently exited training and racing, over a period of 12 months [18]. The exit outcome reported most frequently in that study, was that horses were retired or rehomed, either within the TB industry (19%) as breeding stock or doing industry related work, or outside the TB industry (24%) doing various activities such as pleasure or performance riding, Pony Club, stock work or retired to paddock [18]. A large proportion of study horses (45%) that left racing stables had transitional or temporary destinations reported (spelling, moved to a different trainer, etc.), likely due to the short time frame of interest for the study [18]. This study also found that 2% of the study cohort died after they entered a racing stable and 6% were sent to the abattoir directly from racing [18]. The reasons most commonly reported by Thomson *et*. *al*. for exiting a racing stable in Australia were poor performance (36%) followed by health disorders (31%) and ‘other’ reasons (17%) [18]. Similarly, a New Zealand (NZ) study found only 32% of retirements were associated with injury or illness [19]. A recent study from the United States (US) reported that while off-the-track TB (OTTB) horses or TBs transitioning into a career after racing, were more likely to have musculoskeletal and gastrointestinal issues compared to their non-OTTB counterparts, they were as likely to have resolution of any known issues [20]. Reed *et*. *al*. reported that 97% of the owners of OTTB said they would be open to acquiring another OTTB [20]. A UK study of a cohort of horses followed from birth to four years of age reported that 4% died after entering training, however the reasons for death were not reported [14]. Rules introduced in 2016 by Racing Australia (RA) require the ownership details of a foal to be registered within 60 days of birth (Australian Rules of Racing [AR] AR 34) and the notification of retirement of a horse within one month of the decision to retire (AR 51), in an effort to improve traceability of TBs prior to and after training and racing [21]. However, information on what was occurring prior to the introduction of these rules is needed as a benchmark to assist racing authorities to audit current compliance with rules, and to inform industry endorsed rehoming protocols.

This study investigated the 2010 VIC TB foal crop (VFC) to identify the barriers to transition from stud farm to racetrack, and the outcomes for horses leaving training and racing. The reasons for not entering training and drivers for exiting the racing industry were also investigated. The details of health disorders that prevented horses from entering training or were associated with their exit from the racing industry are described.

## Materials and methods

**Fig 1:**
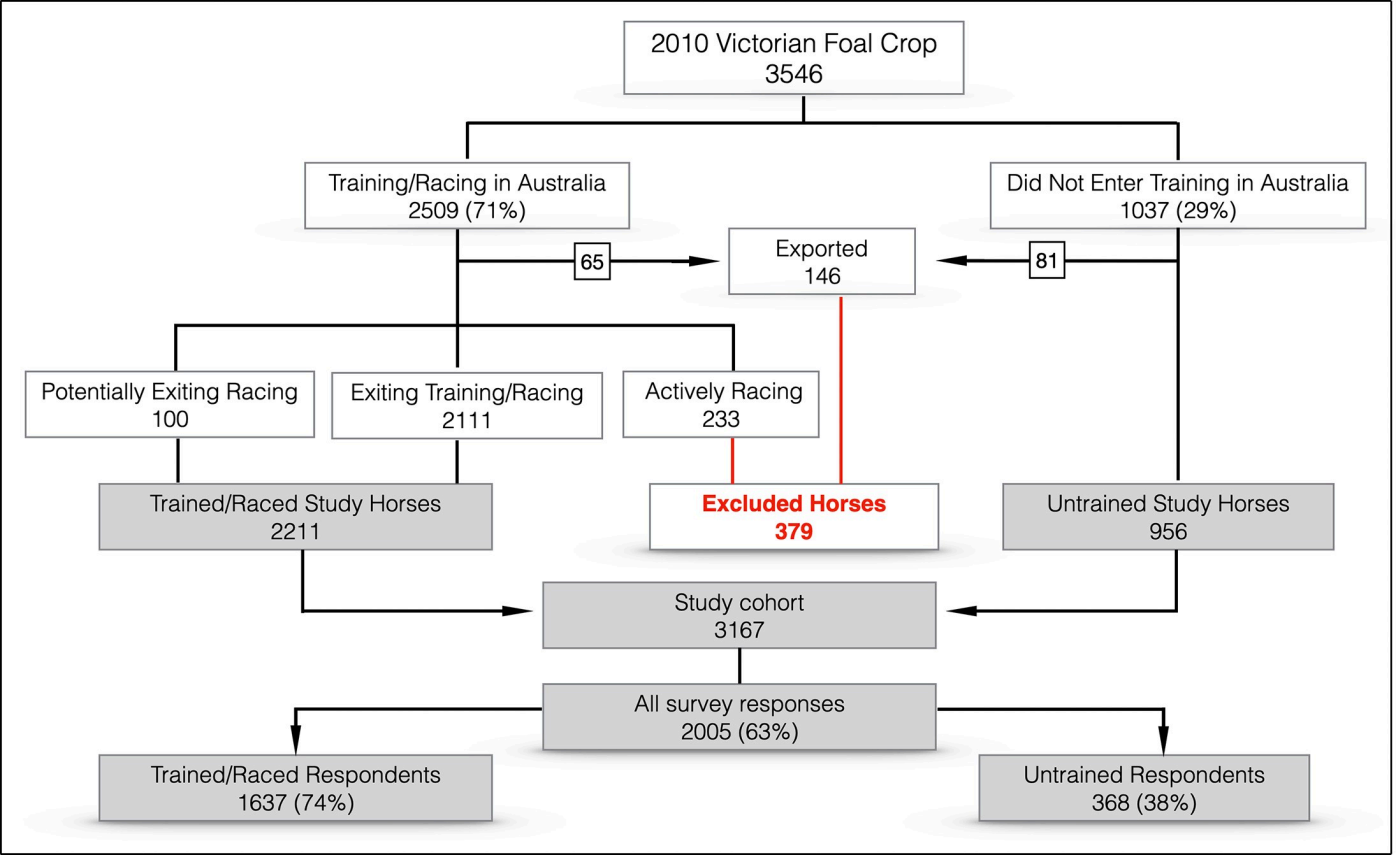
Flow chart of survey enrolment and response rate for the 2010 Victorian foal crop.

The source population for this study comprised the 3546 TB horses of the Victorian foal crop (VFC), identified in a racing performance study [17]. Training and racing records obtained from RA were used to determine the training and racing status for the source population on 1 August 2018. The aim of this study was to determine the age at time of exit, the reason for exit and the outcome for horses that had no record of entering training, or that were no longer actively training and racing in Australia. The study population was comprised of the 3167 horses from the VFC that had either: (1) had no record of entering training (*n* = 956); (2) had entered training but had a non-active status (*n* = 2111); or (3) had an active status on 1 August 2018 but had not trialled or raced between 1 February 2018 and 31 July 2018 (*n* = 100), in RA records. These horses were classified as ‘untrained’, ‘exiting training/racing’ and ‘potentially exiting racing’, respectively (Fig 1). A horse was considered to have officially entered training if a stable return was lodged, the horse had participated in an official trial or jump out, or started in a race. A stable return is a form that records the presence of a horse in a premises, such as the stable of a licensed trainer. An official trial is a practice race over a set distance, run on an official racetrack under the supervision of stipendiary stewards, where horses are not expected to do everything possible to win. A jump out is a training run conducted under the supervision of stewards where the horse starts the run in the starting gates or barriers, but is not required to exercise for a set distance. Horses recorded by RA or the ASB as exported for racing or breeding purposes, or that were still active within Australia on 1 August 2018, and that had participated in a trial or race between 1 February 2018 and 31 July 2018 (*n* = 379) were excluded from enrolment in the study (Fig 1).

### Questionnaire

An online questionnaire was developed for each horse eligible for enrolment into the survey using biographical details from ASB records for untrained horses and RA records for horses that trained or raced. The questionnaire listed each horse’s unique identifier, or life number, registered with the ASB, the horse name, if a name had been registered with RA or the ASB, sire, dam, sex and date of birth (S1 File). The last registered trainer was identified for horses that had raced or trained. The registered breeder was identified for horses that did not officially race or train. These individuals are hereafter referred to as the study participants. Contact details for study participants were provided by the ASB for untrained horses and RA for horses that trained or raced. A personalised email was sent to each study participant on 24 December 2018, listing each of the study participant’s eligible horses for the study and providing a hyperlink to an online questionnaire (S2 File) for each horse. The survey requested the study participant to nominate an outcome for each horse leaving their care, the age of the horse at the time of outcome and a reason for the outcome, if the horse had not entered training or was not currently participating in the racing industry (S1 File). The options for the question requesting the study participant to nominate an outcome, included a category of ‘other’ that could be selected if it was felt the listed categories were not appropriate. When selecting the ‘other’ category, study participants were asked to provide further comments regarding the horse’s outcome. The comments were reviewed and manually re-classified, where appropriate.

Study participants were asked to provide a reason for the outcome for each horse. They were provided with options that were similar to those available on the stable return lodgement forms, such as poor performance; illness or injury; owner request or proactive decision, and behaviour. In addition to the standard stable return options, study participants could select ‘other’ and provide further comment on the specific reason for the nominated outcome. When study participants selected illness or injury as a reason for the outcome, they were asked to provide further information on the specific type of injury or illness.

The study and survey design were approved by the University of Melbourne Human Ethics Advisory Group, Application ID 1748714. All subjects gave their informed consent for inclusion before they participated in the study.

Questionnaires were sent via email to 1,667 study participants. Study participants were requested to complete surveys for 3,167 horses that were selected for enrolment in the study. Each questionnaire was followed up with a phone call, if the study participant had not completed the survey by 31 January 2019. The survey was closed for participants on 29 June 2019.

### Data management and analysis

A custom-designed (FileMaker™ Pro 17 Advanced 17.0.7.700) database was created to link horse records, training and racing records, and contact details of study participants. Analysis of survey responses was carried out using R version 3.6.1 (R Core Team, 2019).

The results for this study are reported as counts and percentages for each category. Survey outcomes of trained/unraced horses and trained/raced horses are detailed separately in the tables but are reported under a collective heading of ‘trained horses’ unless otherwise stated. Survey responses categorising horses as actively training/racing, active non-stable training and spelling are aggregated in the table under a group heading of ‘participating in racing’, but are reported separately in the text. Spelling was considered to be a temporary exit from racing, due to respondents indicating the horse’s likelihood to return to racing. Active non-stable training is a category of training status used on industry stable return lodgement forms, to indicate the horse is undertaking some form of training at a premises other than a licensed trainer’s stable. The type of activities that are expected for this category are pre-training or rehabilitation, for example, at a facility with a water walker. A water walker is a machine where the horse walks around in a loop that is partially filled with water. Horses that had a retirement outcome as a lead pony or clerk of the course horse, were collectively categorised as an ‘industry’ retirement. A lead pony is a horse that assists in the training of horses, but does not actively compete in races or trials. A clerk of a course horse, is a horse used by outriders at the races, to help lead a horse to the barriers or starting gates, help catch any horses that lose their rider before or during a race, or assist with returning horses to the mounting yard after the race.

Injuries identified as musculoskeletal injuries that were unable to be classified as a fracture or a tendon/ligament injury, were reported in the table as musculoskeletal—other with further detail provided in the text. Injuries reported as wounds/trauma were injuries described by respondents as traumatic incidents resulting in non—musculoskeletal or unspecified injuries involving fences and gate posts, fights with other horses, dog attacks or burns inflicted during a bush fire. Comparisons of proportions between sex and training groups were carried out using the chi-squared test with the probability of making a Type 1 error set to 0.05.

### Variation in response rate between untrained and trained horses

The likelihood of misclassification of horse outcome status (deceased, not deceased) was acknowledged as a potential problem in this study because, for some horses, a substantial period of time may have elapsed between the date of the outcome and the date the survey was carried out. Due to this delay, there was an increased likelihood that study participants might misclassify their horse’s outcome due to poor recall [22]. To address this issue a probabilistic sensitivity analysis was carried out using methods described previously by Lash *et*. *al*. [23]. In brief, we sought to determine how much error there would need to be in assignment of outcome status to produce a biologically meaningful change in the proportion of untrained horses that left the racing industry due to death.

*A priori* we assumed the sensitivity and specificity for correctly assigning outcome status for trained horses was 0.90 and 0.99, respectively. This meant that, for horses that had entered training, there was a 90% chance that a study participant would correctly assign a horse as having died and a 99% chance that that a study participant would correctly assign a horse as alive. If a horse was alive it was likely that it was still under the care of the study participant, justifying our assumption of a relatively high specificity of 99%. In the same manner, the sensitivity and specificity for correctly assigning outcome status for untrained horses was set to 0.80 and 0.90, respectively. Correct assignment of sensitivity and specificity for untrained horses were assumed to be less than that of trained horses due to the greater period of time that had elapsed between the pre-training period and the date on which the questionnaire was completed.

## Results

A total of 3,167 questionnaires were sent to 1,667 study participants for horses enrolled in the survey. Responses were received for 2,011 questionnaires, and six questionnaires were excluded from analysis due to data incompatibilities, such as a gelding being nominated as an ASB broodmare. After excluding these horses, 2,005 of the 3,167 questionnaires were retained for analysis (63% response rate, Fig 1). Data from RA and ASB for horses that were deceased (30) or were retired as ASB Bloodstock (17), were used to provide answers for a small number (47 of 2,005) of surveys. Survey responses were returned for 1052 female and 953 male horses.

Responses were not received for 1,156 (37%) study horses. These non-respondents could be divided into several groups: 487 horses where the study participant could not be reached and messages were left using their contact number; 279 horses where the survey participant could not be reached due to an incorrect email address, disconnected or incorrect phone numbers, they were deceased, or disqualified from training; 163 horses where the only contact detail available was an email address; 86 questionnaires where study participants indicated they would respond but had not completed the survey by the closing date and 73 horses where the study participant could not be reached and there was no option to leave a message. Study participants for 68 (2% of 3,167) horses chose to opt out of the survey.

### Untrained horses

Of the 3167 horses included in the survey, 956 horses had no record with RA of entering training and responses were received for 38% (*n* = 368) of these horses.

#### Untrained horses: Deceased

Of the 368 survey responses for untrained horses, death (34%) was the most frequent outcome reported. Four of these 125 survey responses identified that the horses were sent directly to an abattoir (Table 1), due to lack of available feed (*n* = 3) or dangerous behaviour (*n* = 1). The age of death was reported for 86% (108 of 125) of responses. Eighteen percent of deaths in untrained horses were reported to have occurred at or before the horse was 6 months of age, the age by which most horses would have been weaned from their dams. Overall, the median age of death of untrained horses was one year of age (Q1 [quartile 1] 0.8; Q3 [quartile 3] 2).

**Table 1:** Reported outcomes for the Victorian 2010 cohort.

Outcome	Untrained horses *n* (%)	Trained/ unraced horses *n* (%)	Trained/ raced horses *n* (%)	Male horses *n* (%)	Female horses *n* (%)	Total horses *n* (%)
Retired/rehomed	101 (27)	161 (66)	1,049 (75)	580 (61)	731 (70)	1311 (65)
Deceased	125 (34)	33 (14)	164 (12)	179 (19)	143 (14)	322 (16)
Sold^a^	85 (23)	10 (4.1)	22 (1.6)	50 (5.2)	67 (6.4)	117 (5.8)
Participating in racing	9 (2.4)	2 (0.8)	62 (4.5)	51 (5.4)	22 (2.1)	73 (3.6)
Returned to owner	1 (0.3)	10 (4.1)	28 (2.0)	19 (2.0)	20 (1.9)	39 (1.9)
Transferred	1 (0.3)	2 (0.8)	11 (0.8)	9 (0.9)	5 (0.5)	14 (0.7)
Other	1 (0.3)	1 (0.4)	1 (0.1)	1 (0.1)	2 (0.2)	3 (0.1)
Unknown/unspecified	45 (12)	25 (10)	56 (4.0)	64 (6.7)	62 (5.9)	126 (6.3)
Total^b^	368 (100)	244 (100)	1,393 (100)	953 (100)	1,052 (100)	2,005 (100)

^a^ Includes one horse exported to China

^b^ Subject to rounding error.

Study participants were asked to describe the circumstances of death. The majority of reported deaths (75%) in untrained horses were not associated with any form of exercise, such as training or pretraining. However, training/pre-training accounted for 9% of deaths in this group, despite these horses having no official record of training. The circumstances of death for the remainder were unspecified. Musculoskeletal injuries were the most frequently reported health disorders resulting in death for untrained horses. Musculoskeletal—fracture injuries were the most frequently reported (28%), followed by soft tissue wounds/trauma and digestive disorders (Table 2).

**Table 2:** Illnesses and injuries as cause of death for the Victorian 2010 cohort.

Illness or injury	Untrained horses *n* (%)	Trained/ unraced horses *n* (%)	Trained/ raced horses *n* (%)	Total horses *n* (%)
Musculoskeletal—fracture	27 (28)	18 (67)	60 (44)	105 (40)
Musculoskeletal—tendon/ligament	10 (10)	2 (7.4)	16 (12)	28 (11)
Musculoskeletal—other	3 (3.1)	0 (0.0)	3 (2.2)	6 (2.3)
Wounds/trauma	16 (17)	2 (7.4)	11 (8.1)	29 (11)
Digestive disorder	12 (12)	3 (11)	12 (8.8)	27 (10)
Sudden death	8 (8.2)	1 (3.7)	8 (5.9)	17 (6.5)
Cardiac or metabolic disorder	2 (2.1)	3 (11)	11 (8.1)	16 (6.2)
Lower respiratory tract disorder	3 (3.1)	1 (3.7)	4 (2.9)	8 (3.1)
Congenital disorder	8 (8.2)	0 (0.0)	0 (0.0)	8 (3.1)
Infection	4 (4.1)	0 (0.0)	3 (2.2)	7 (2.7)
Reproductive/birth trauma	0 (0.0)	0 (0.0)	4 (2.9)	4 (1.5)
Immune disorder	0 (0.0)	0 (0.0)	1 (0.7)	1 (0.4)
Other disorder	1 (1.0)	0 (0.0)	1 (0.7)	2 (0.8)
Unknown/unspecified	7 (7.2)	0 (0.0)	5 (3.7)	12 (4.6)
Total	101^a^	30^b^	139^c^	270^d^

^a^ 101 responses were received from 97 questionnaires due to one questionnaire giving three responses (fracture, tendon or ligament injury and wounds/trauma) and two questionnaires giving two responses (tendon or ligament injury and wounds/trauma [*n* = 1]); fracture and tendon or ligament injury [*n* = 1])

^b^ 30 responses from 27 questionnaires as one questionnaire gave three responses (fracture, tendon or ligament injury and wounds/trauma) and one gave two responses (fracture and wounds/trauma)

^c^ 139 responses from 136 questionnaires due to three questionnaires giving two responses (fracture and wounds/trauma [*n* = 1]; fracture and tendon or ligament injury [*n* = 1]; and tendon or ligament injury and wounds/trauma [*n* = 1])

^d^ 270 responses from 260 questionnaires due to multiple disorders describe per horse for eight questionnaires.

#### Untrained horses: Retired or rehomed

Horses categorised as retired or rehomed were the second most frequent (27%) outcome for untrained horses (*n* = 101, Table 1). These horses were either retained within the TB industry as ASB breeding stock (*n* = 16: 15 females, one male), or were rehomed outside the TB industry (*n* = 85, Table 1). Equestrian or pleasure pursuits (43%) were the most frequent retirement outcome for horses rehomed outside the TB industry, followed by unridden/companion pursuits and becoming broodmares for foals not intended for racing, in descending order (Table 3). When the reasons for retirement were investigated, health disorders were the most frequent reason reported (24%), followed by poor performance (despite the horses having no official record of training). The remainder of the known reasons were owner request, other and unsuitable behaviour in descending order of frequency (Table 4). A variety of health disorders were listed as reasons for retirement. The most frequent health disorder associated with untrained horses retiring or being re-homed was musculoskeletal—tendon/ligament injuries (Table 5). Of the 16 horses that had ‘other’ as the reason for retirement seven of these were due to financial reasons, four were ‘never intended to race’, three were ‘too small’, one was too old before the decision was made to enter training and one was not eligible to race due to a parentage DNA discrepancy. Age of retirement was provided for 61% (62 of 101) of untrained horses, with a median age of three (Q1 2, Q3 4) years. Untrained horses categorised as equestrian or pleasure horses were reported to have participated in a wide range of activities in their post racing careers (Table 6) with the most frequent ridden pursuit being a pleasure horse or hack.

**Table 3:** Outcomes for retired or rehomed horses from the 2010 Victorian cohort.

Outcome	Untrained horses *n* (%)	Trained/ unraced horses *n* (%)	Trained/ raced horses *n* (%)	Male horses *n* (%)	Female horses *n* (%)	Total horses *n* (%)
Equestrian/pleasure	43 (43)	59 (37)	509(49)	403 (70)	208 (29)	611 (46)
ASB Bloodstock	16 (16)	48 (30)	285 (27)	5 (0.9)	344 (47)	349 (27)
Companion/unridden	24 (24)	15 (9.3)	78 (7.4)	65 (11)	52 (7.1)	117 (8.9)
Returned to owner	3 (3.0)	23 (14)	58 (5.5)	47 (8.1)	37 (5.1)	84 (6.4)
Non-TB broodmare	6 (5.9)	6 (3.7)	47 (4.5)	0 (0)	59 (8.1)	59 (4.5)
Industry	0 (0.0)	1 (0.6)	14 (1.3)	14 (2.4)	1 (0.1)	15 (0.1)
Other	1 (1.0)	1 (0.6)	2 (0.2)	4 (0.7)	0 (0)	4 (0.03)
Unknown/unspecified	8 (7.9)	8 (5.0)	61 (5.8)	44 (7.6)	33 (4.5)	77 (5.9)
Total	101	161	1054^a^	582^b^	734^c^	1,316^d^

^a^ 1,054 responses from 1,049 questionnaires as five questionnaires gave two responses (broodmare for non-TBs and equestrian/pleasure pursuits (*n* = 2; female), industry and equestrian/pleasure pursuits (*n* = 2; male) and ASB broodmare and broodmare for non-TBs (*n* = 1)

^b^ 582 responses from 580 questionnaires as two questionnaires gave two responses

^c^ 734 responses from 731 questionnaires as three questionnaires gave two responses

^d^1,316 responses from 1,311 questionnaires as five questionnaires gave two responses.

**Table 4:** Reasons for rehoming or retirement for the 2010 Victorian cohort.

Reasons for retirement	Within TB industry *n* (%)	Outside TB industry *n* (%)	Untrained horses *n* (%)	Trained/ unraced horses *n* (%)	Trained/ raced horses *n* (%)	Total retirement. *n* (%)
Poor performance	109 (30)	448 (47)	20 (20)	78 (48)	459 (44)	557 (42)
Illness or injury	98 (27)	270 (29)	24 (24)	37 (15)	307 (29)	368 (28)
Owner request	84 (23)	112 (12)	16 (16)	12 (7.5)	168 (16)	196 (15)
Behaviour	9 (2.5)	30 (3.2)	5 (5.0)	6 (3.7)	28 (2.7)	39 (3.0)
Other	3 (0.8)	30 (3.2)	16 (16)	1 (0.6)	16 (1.5)	33 (2.5)
Unknown/unspecified	61 (17)	62 (6.5)	20 (20)	27 (17)	76 (7.2)	123 (9.4)
Total	364	952^a^	101	161	1054^b^	1316^c^

^a^ 952 responses from 947 questionnaires

^b^ 1,054 responses from 1,049 questionnaires and

^c^ 1,316 responses from 1,311 questionnaires; due to five questionnaires giving two reasons each (illness or injury and poor performance [*n* = 2] and poor performance and owner’s decision [*n* = 3])

**Table 5:** Illness or injuries leading to retirement, stratified by retirement within or outside the TB industry and by training level.

Illness or injury	Within TB industry *n* (%)	Outside TB industry *n* (%)	Untrained horses *n* (%)	Trained/ unraced horses *n* (%)	Trained/ raced horses *n* (%)	Total retirement *n* (%)
Musculoskeletal—tendon/ligament	30 (31)	98 (43)	7 (29)	11 (30)	110 (36)	128 (35)
Musculoskeletal—other	25 (26)	66 (24)	2 (8.3)	11 (30)	78 (25)	91 (25)
Musculoskeletal—fracture	23 (24)	29 (11)	2 (8.3)	5 (14)	45 (15)	52 (14)
Upper respiratory tract disorder	5 (5.1)	30 (11)	2 (8.3)	3 (8.1)	30 (9.8)	35 (9.5)
Lower respiratory tract disorder	3 (3.1)	18 (6.7)	1 (4.2)	1 (2.7)	19 (6.2)	21 (5.7)
Wounds/trauma	4 (4.1)	6 (2.2)	4 (16.7)	1 (2.7)	5 (1.6)	10 (2.7)
Poor conformation	1 (1.0)	7 (2.6)	2 (8.3)	3 (8.1)	3 (1.0)	8 (2.2)
Congenital disorder	0 (0.0)	4 (1.5)	1 (4.2)	1 (2.7)	2 (0.7)	4 (1.1)
Digestive disorder	0 (0.0)	3 (1.1)	0 (0.0)	2 (5.4)	1 (0.3)	3 (0.8)
Cardiac/metabolic disorder	0 (0.0)	1 (0.4)	0 (0.0)	0 (0.0)	1 (0.3)	1 (0.3)
Immune disorder	1 (1.0)	0 (0.0)	1 (4.2)	0 (0.0)	0 (0.0)	1 (0.3)
Other disorder	0 (0.0)	4 (1.5)	3 (13)	0 (0.0)	1 (0.3)	4 (1.1)
Unknown/unspecified	7 (7.1)	7 (2.6)	0 (0.0)	0 (0.0)	14 (4.6)	14 (3.8)
Total	99^a^	273^b^	25^c^	38^d^	309^e^	372 ^f^

^a^ 99 responses from 98 questionnaires because one ASB horse retired due to both a fracture and an upper respiratory tract disorder

^b^ 273 responses from 270 questionnaires due to three questionnaires giving two responses (fracture and an upper respiratory tract disorder [*n* = 1]), tendon/ligament injury and wounds/trauma (*n* = 1) and an upper respiratory tract disorder and unspecified joint disorder (*n* = 1)

^c^ 25 responses from 24 questionnaires due to one questionnaire giving two responses (tendon or ligament injury and wounds/trauma)

^d^ 38 responses from 37 questionnaires due to one questionnaire giving two responses (upper respiratory tract disorder and fracture)

^e^ 309 responses from 307 questionnaires due to two questionnaires giving two responses (upper respiratory tract disorder and fracture [*n* = 1] and upper respiratory tract disorder and unspecified joint disorder (musculoskeletal–other) [n = 1])

^f^ 372 responses from 368 surveys.

**Table 6:** Frequency of specific disciplines in horses retired to equestrian/pleasure pursuits.

Equestrian pursuit	Untrained horses *n* (%)	Trained/ unraced horses *n* (%)	Trained/ raced horses *n* (%)	Male horses *n* (%)	Female horses *n* (%)	Total Equestrian *n* (%)
Pleasure horse/hack	18 (41.9)	31 (52.5)	212 (41.7)	178 (44.2)	83 (39.9)	261 (42.7)
Show jumping	7 (16.3)	5 (8.5)	64 (12.6)	49 (12.2)	27 (13.0)	76 (12.4)
Eventing	3 (7.0)	1 (1.7)	69 (13.6)	60 (14.9)	13 (6.3)	73 (12.0)
Adult riding	7 (16.3)	7 (11.9)	45 (8.8)	36 (8.9)	23 (11.1)	59 (9.7)
Pony Club	3 (7.0)	8 (13.6)	48 (9.4)	37 (9.2)	22 (10.6)	59 (9.7)
Dressage	2 (4.7)	2 (3.4)	54 (10.6)	36 (8.9)	22 (10.6)	58 (9.5)
Show horses	4 (9.3)	3 (5.1)	33 (6.5)	27 6.7)	13 (6.3)	40 (6.5)
Stock horses	3 (7.0)	4 (6.8)	14 (2.8)	9 (2.2)	12 (5.8)	21 (3.4)
Polo	0 (0.0)	4 (6.8)	13 (2.6)	3 (0.7)	14 (6.7)	17 (2.8)
Riding for the Disabled	0 (0.0)	0 (0.0)	3 (0.6)	3 (0.7)	0 (0.0)	3 (0.5)
Rodeo	0 (0.0)	0 (0.0)	3 (0.6)	1 (0.2)	2 (1.0)	3 (0.5)
Unknown/unspecified	1 (2.3)	4 (6.8)	38 (7.5)	32 (7.9)	11 (5.3)	43 (7.0)
Total	48^a^	69^b^	596^c^	471^d^	242 ^e^	713 ^f^

^a^ 48 response from 43 questionnaires due to two horses categorised as participating in multiple disciplines

^b^ 69 responses from 59 questionnaires due to five horses categorised as participating in multiple disciplines

^c^ 596 responses from 509 questionnaires due to 49 horses categorised as participating in multiple disciplines

^d^ 471 responses from 403 questionnaires due to 37 horses categorised as participating in multiple disciplines

^e^ 242 responses from 208 questionnaires due to 19 horses categorised as participating in multiple disciplines

^f^ 713 responses for 611 questionnaires due to 56 horses categorised a participating in more than one discipline.

#### Untrained horses: Sold

Sale was the third most frequent outcome described by respondents for untrained horses, with no further information provided by the study participant (Table 1). The reason for sale was infrequently given with 67% (57 of 85) unknown or unspecified. The next most frequent response was owner request (20%, 17 of 85), followed by behaviour (*n* = 3), injury/illness (*n* = 2). One horse was sold because it was too small for racing and one due to the participant losing their property. The two responses that reported horses sold due to injury reported a laceration injury to a leg considered limiting to the horse’s future racing career and an angular limb deformity that made the horse unsuitable for racing. The former horse was sold as a riding horse. Of the 85 horses that were categorised as sold at a public or private sale, 57% (*n* = 49) were recorded on official TB industry sales records or by survey respondents as being sold at a yearling or weanling sale. The median age of sale for untrained horses was one (Q1 1; Q3 2) year.

#### Untrained horses: Participating in the racing industry

Though the horses enrolled in the study had no official industry record of training or racing, seven horses were categorised as actively training or racing (four females and three males) and two as spelling (two females) during the 2018–2019 racing season.

#### Untrained horses: Returned to owner, transferred, and other

For the outcome categories of returned to the owner, transferred to another trainer and other (financial dispute), the survey participant was unable to provide any further information (Table 1).

### Trained; unraced and raced horses

Of the 2,211 horses who had an industry record of entering training, responses were received for 74% (*n* = 1,637) surveyed horses that had entered training. The two most frequent outcomes for horses that trained but were unraced (*n* = 244) and horses that trained and raced (*n* = 1,393), were retired and deceased, as these outcomes accounted for the vast majority of these groups they were therefore collapsed into a single group of ‘trained horses’ (*n* = 1,637). Where differences exist between these two groups they are reported separately in the text.

#### Trained horses: Retired or rehomed

Retirement or being rehomed was the most frequent outcome for trained horses at the end of their career in the racing industry (Table 1), accounting for nearly three quarters (74%, 1,210 of 1,637) of the survey responses for trained horses (trained/unraced, *n* = 161; trained/raced, *n* = 1,049). While trained horses were most frequently rehomed outside the TB industry (71% 862 of 1,210), more than a quarter of all trained horses remained within the TB industry, either as ASB bloodstock (28%, 333 of 1,210) or as a clerk of the course or lead pony (1%, 15 of 1,210) (Table 3). For the 862 horses retiring outside of the TB industry, 66% (*n* = 568) were categorised as going on to equestrian or pleasure riding outcomes. The next most common retirement outcomes were companion/unridden pursuits (11%, *n* = 93), returned to owner with no further information available (9%, *n* = 81), broodmare for non-TBs (6%, *n* = 53) and other (0.3%, *n* = 3). For the remaining 8% (*n* = 69) the respondent was unable to categorise retirement outcome (Table 3).

When stratified by sex, males were more likely than females to be re-homed outside the TB industry (χ^2^ (1, N = 1,210) = 98.65; p <0.01) or used as a lead pony or clerk of the course horse (χ^2^ (1, N = 1,210) = 14.15; p < 0.01) than females. Females were more likely to be retired as ASB breeding stock (χ^2^ (1, N = 1,210) = 12.71; p <0.01). There was no significant difference between training/racing strata, for horses retiring within or outside the TB industry (χ^2^ (1, N = 1,210) = 0.478; p 0.49). The age of retirement was provided for 91% of trained/unraced horses and 96% of trained/raced horses. The median age of retirement was remarkably consistent at five (Q1 4; Q3 6) years and did not vary by sex, nor for horses that started racing at two, three or four-years of age. When data were stratified by the highest level of training, the median age of retirement for trained/unraced horses was four (Q1 3; Q3 5) years and trained/raced horses was five (Q1 4; Q3 6) years. There was a slight increase in the age of retirement to six (Q1 5; Q3 7) years, for horses that retired to become lead ponies or clerk of the course horses.

The reason for retirement was provided for 91% (1,107 of 1,210) of retired or rehomed horses (Table 4). The majority of retirements were considered voluntary, due to reasons such as poor performance (44%) or at the owner’s request (15%,) followed by involuntary reasons for retirement, illness or injury, behaviour and other (Table 4). It should be noted that comments associated with poor performance and owner request categories were similar, with ‘reached competitive limit, ‘lost interest in racing’, ‘lacking ability’ and ‘form tapering with age’ provided for both reasons. This suggests that study participants were using these two categories interchangeably.

When responses were stratified by whether the horses retired within (*n* = 348) or outside the TB industry (*n* = 862), a greater proportion of horses that retired within the TB industry retired due to owner request, than horses that retired outside the TB industry (χ^2^ (1, N = 1,107) = 37.07; p < 0.01). Conversely, the proportion of horses retired or rehomed due to poor performance, was greater for horses retiring outside the TB industry (χ^2^ (1, N = 1,107) = 20.14; p < 0.01). Forty eight percent (166 of 344) of horses that were retired due to injury or illness were categorised as equestrian/pleasure horses, suggesting the presence of a health disorder that led to their retirement, did not prevent them from subsequently pursuing a second career as a riding horse. Musculoskeletal injuries were the most frequently reported health disorder resulting in retirement for trained horses, with musculoskeletal–tendon/ligament injuries (35%, 121 of 344) the most frequently specified health disorder, followed by musculoskeletal—other injuries and fractures (Table 5). The musculoskeletal—other injuries reported in 89 responses, described musculoskeletal injuries that were not able to be classified as fracture or tendon/ligament injury. Forty-four responses describe health disorders associated with joint problems such as, degenerative joint disease (*n* = 7), osteochondritis dissecans (*n* = 3), and unspecified joint problems (*n* = 34). Thirteen responses described an unspecified lameness. Eight responses described disorders of the neck, back or pelvis. Ten horses retired due to disorders of the hoof and six retired due to muscle disorders. The remaining responses were miscellaneous singular health disorders.

There was no significant difference in the proportion of trained and untrained horses, categorised as retired or rehomed due to injury or illness (χ^2^ (1, N = 2,005) = 1.01; p = 0.316). The comments provided for horses retiring due to behaviour, cited poor barrier behaviour and embargoes, as the most frequent behavioural complaint. Under the AR if a horse’s behaviour is deemed to be unsafe in or around the barriers, the horse will be prevented from racing, or an embargo placed on the horses’ racing status. The trainer must subsequently demonstrate that the horse can safely enter, stand and leave the barriers before being allowed to race again.

Of the 17 responses where study participants selected ‘other’ as a reason for retirement or re-homing, 12 reported the horse as being too old, four were retired for financial reasons, and one horse retired due to trainer disqualification. Similar to the untrained horses the most frequent ridden pursuit was as a pleasure horse.

#### Trained horses: Deceased

Death (*n* = 197, 12%) was the second most frequently identified outcome for trained horses. When stratified by the level of training, the median age of death was three (Q1 3; Q3 5) years for trained/unraced horses and five (Q1 4; Q3 6) years for trained/raced horses. Of these 197 responses (Table 1), 13 identified that the horse was sent to an abattoir (one trained/unraced and 12 trained/raced); five due to injury or illness, two due to injury and behaviour, three for behaviour only, two due to poor performance and one at the owner’s request. For the trained/unraced horses categorised as deceased (*n* = 33) death was most frequently associated with training or pretraining (39%, 13 of 33) followed by non-exercising (24%, 8 of 33) and during a trial or jump out (15%, 5 of 33), with the remaining circumstances unspecified. For the 164 deceased trained/raced horses, the most frequent circumstance of death was non-exercising (42%, 68 of 164) followed by death during a race (29%, 47 of 164) and during training/pre-training (16%, 26 of 164), with two deaths reported during a trial or jump out and 21 responses where the circumstances were not specified.

Musculoskeletal injuries were the most frequently reported health disorder resulting in death for trained horses. Fracture was the health disorder reported most frequently as a cause of death, accounting for 48% of deaths in all trained horses, followed by tendon or ligament injuries, digestive, and cardiac disorders (Table 2). Horses that had trained but were unraced had a higher proportion of fracture and cardiac disorders than trained/raced horses (Table 2). Untrained horses were more likely than trained horses to be categorised as deceased (χ^2^ (1, N = 2,005) = 107.22; p <0.001). There was no significant difference in the proportion of horses categorised as deceased between the trained/ unraced and trained/raced horses (χ^2^ (1, N = 2,005) = 0.60; p 0.438). Males were more likely to be deceased than females in the trained horse group (χ^2^ 1, N = 1,637) = 9.99; p 0.01).

#### Trained horses: Sold, exported, returned to owner or transferred to another trainer

A small proportion of trained horses were classified as being sold at a public or private sale (2%, 31 of 1637), transferred to another trainer (0.8%, 13 of 1,637) or returned to owner (2%, 38 of 1,637). The responses to these surveys did not provide further details regarding the current activity of these horses. There was one additional mare categorised as exported (destination China) with no reason for export provided, therefore, that response was combined with horses categorised as sold for ease of reporting bringing the total sold/exported to 32 trained horses (Table 1). The age of sale was provided on 24 surveys with a median age of sale of five (Q1 4; Q3 6) years. The reason for sale was given for 22 of the 32 horses. The most frequently reported reason for sale was poor performance (*n* = 15) followed by injury (*n* = 4), unsuitable behaviour (*n* = 1), owner’s decision (*n* = 1) and being too old for racing (*n* = 1). The specific injuries identified by respondents as a reason for sale were musculoskeletal—tendon/ligament injuries (*n* = 2), an eye injury and a horse that was a bleeder (exercise induced pulmonary haemorrhage). Twelve of the 38 responses categorised as ‘returned to owner’ specified a reason the horse left the study participant’s care. Seven were due to poor performance, two were returned to the owner due to a health disorder, one due to behavioural issues, one due to financial reasons and another due to personal reasons. These transitional categories represent a loss of current information about the horse once it leaves the care of the study participant (Table 1).

#### Trained horses: Participating in the racing industry

There were 64 horses (48 males and 16 females) categorised as participating in the 2018–2019 racing season, either actively training and racing (*n* = 47), doing non-stable training such a pre-training or exercising at a water walker (*n* = 3) or spelling (*n* = 14) (Table 1). Males were more likely to still be actively participating in racing during the 2018–2019 racing season compared with females (χ^2^ (1, N = 1,637) = 12.57; p <0.01).

### Variation in response rate

When stratified by training status, the response rate was lower for horses that had no official record of entering training in Australia. Study participants associated with untrained horses (326 of 584) were more likely to have incorrect contact details (*n* = 185), or to only have an email address as their contact details (*n* = 141) which prevented them from being followed up by phone, compared to participants associated with trained or raced horses (116 of 572, χ^2^ (1, N = 1,156) = 154.57; p < 0.01). Though a low number of study participants opted out of taking the survey, those study participants were more likely to be associated with untrained horses (40 of 956) than trained horses (28 of 2,211, χ^2^ (1, N = 3,167) = 27.04; p = < 0.01).

The exclusion of 233 horses that were alive and actively training or racing, may have introduced selection bias, resulting in underestimation of the proportion of horses actively participating in racing and overestimating the proportion of horses that were deceased or retired. Of the 100 horses that were enrolled in the study that had an active status, but had not trialled or raced in the six months prior to enrolment (potentially exiting, Fig 1), 85 questionnaires were returned. Forty-eight percent of those questionnaires categorised horses as actively racing or training (*n* = 35) or spelling with the intention to return to racing (*n* = 6).

For our probabilistic sensitivity analyses, a 5% change in the proportion of untrained horses estimated to have been deceased did not occur until the sensitivity for correctly assigning outcome status for untrained horses was reduced to 0.71. We conclude that at least 29 out of every 100 survey responses would need to have been misclassified to result in a five percent change in the estimated proportion of horses classified as deceased.

## Discussion

The most frequent outcome for horses that had trained or raced in Australia according to our study was that they were retired or rehomed (74%) within or outside the TB industry. The proportion of horses reported as retired in this study, was substantially greater than the 40% reported by Thomson *et*. *al*. in 2014 [18]. This difference may be due to the longitudinal nature of this study, compared with the approach used by Thomson *et*. *al*., where the respondents were asked to identify the outcome for the last five horses that left their stable [18]. The current study reported the outcomes for horses from birth until they were eight-years-old, which may explain why more horses had retired from racing. Voluntary retirement (59%) due to poor performance or owner request for trained horses in this study was slightly lower than a NZ study of horses in training over 36 months [19]. This is likely to be due to the increased number of categories used in the current study, to describe the reason for retirement. It is worth noting that poor performance and illness or injury are not necessarily mutually exclusive and qualitative research is needed to more deeply investigate these distinctions. Responses categorising the reason for exit as behaviour or ‘other’, were more likely to be associated with involuntary retirement. Unsuitable barrier behaviour was the most frequent behavioural issue identified by participants, including embargoes on racing. Horses that retired due to ‘other’ reasons, were described as being too old or that the owner was financially unable to keep the horse racing. Tendon and ligament injuries were the most frequent health disorder, for horses categorised as retired or rehomed, for both untrained and trained horses. The voluntary retirement of trained horses at a median of five-years of age in this study was remarkably consistent, and suggests that industry-level or horse performance effects, rather than biological effects are the predominate driver of retirement for this group. This finding is supported by recent Australian research that found the majority of horses had their last race start at by five-years of age [17].

Interestingly, although 28% of trained horses retired due to injury, nearly half of those injured horses were categorised as subsequently participating in ridden pursuits, such as equestrian or pleasure horse activities. This suggests that while the injury may have made the horse unsuitable for racing, it did not prevent the horse from undertaking less intensive ridden pursuits in their post-racing career. The most frequent health disorders reported for retired horses were tendon or ligament injuries and non-fracture related musculoskeletal injuries. These findings are supported by a recent US study, that reported that while OTTB were more likely to have musculoskeletal and gastrointestinal disorders, these conditions were as likely to resolve, as the same conditions in non-OTTB controls [20]. Further research is needed to investigate the length of time that horses continue to participate in these post racing careers and what health disorders prevent a successful transition.

Our study found that the greatest barrier to entering training was death, due to injury or illness. The most frequent injuries reported for horses categorised as deceased were fractures and wounds/trauma. Recent Australian research that investigated why TBs failed to transition from the stud farm to the racetrack also found that fractures were the most frequent injury resulting in death [24]. As the median age of death in this group was one year of age, and the deaths were not associated with training, it was most likely that these deaths occurred on the stud farm, before the horse entered training and racing. This agrees with an Australian study investigating TBs that failed to enter training before four years of age, that found that the period of greatest risk of mortality was in the first 12 months of life [24]. Another recent Australian report found that nearly three-quarters of TB foals successfully transition from the stud farm to officially enter training [17]. Further research is needed to investigate the specific risk factors associated with these traumatic injuries to improve outcomes for untrained horses. This would ideally make more horses available for racing and reduce breeding requirements.

Only one percent of trained horses (13 of 1,637) were reported to have been sent to the abattoir, substantially less than the six percent reported by Thomson *et*. *al*. [18]. However, while the number of horses sent to abattoir in this study was lower, the overall percentage of trained horses that died (12%) was higher than the 8% reported by Thomson *et*. *al*. [18]. This may be due to the increased length of the study period, and to the inclusion of deaths occurring during training and racing. Study design may have also influenced the reporting of horses being sent to abattoir, with participants reluctant to report a specific horse as sent to abattoir. This is acknowledged as a limitation in the current study design. The proportion of deceased raced horses (12%) was twice that reported by Wilsher *et*. *al*. for horses racing in the UK prior to four years of age [14]. Again, the length of follow up to eight years of age is likely to have resulted in the increased proportion of deceased horses, with the median age of death for raced horses in the current study being five years. It was an interesting observation that males were more likely to die than females, however the dataset provided for this study did not allow further investigation of risk factors. There are many factors that may confound this observation, including the proportions that raced and trained compared to females, career duration and racing opportunity. However, this study was unable to examine these risk factors and further research is required. The exclusion of horses that were actively training and racing in the six months prior to enrolment into the study, may have also resulted in an overestimation of some of the outcome categories for trained horses.

The third most frequent outcome for untrained horses were horses categorised as sold. The majority of these were sold as weanlings or yearlings at TB industry sales, as racing prospects. The sale of horses to persons not licensed or registered with RA, limited the traceability of horses prior to the horse entering training and again after the horse exited the racing industry. The 2016 introduction of rules, addressing foal registration (AR 34), location of unnamed horses (AR 50), ownership transfer records (AR 34, AR 50), the death of TBs registered with RA (AR 52) and location of horse upon retirement (AR 51), is likely to address this gap [21]. The study participants were unable to describe the horse’s current activities if it had been returned to the owner or transferred to another trainer. Prior to the 2016 rule introductions, only trainers were able to report a horse as retired (AR 51) [21]. The introduction of these rules, not only expanded the reporting requirement to include a specific address of retirement, but it also allowed owners and syndicate managers to record a horse’s retirement details [21]. The effectiveness of these rule changes in reducing gaps in traceability has not yet been reported. Data on horse movement prior to entering training and after exiting the racing industry should be collected and audited, to evaluate whether further traceability gaps exist. As nine horses with no official record of training, were reported to be participating in the racing industry in the 2018–2019 racing season, combined with 20 untrained horses reported as retiring due to poor performance and 11 untrained horses whose circumstance of death were during training/pre-training, this study identified that official records underestimate the number of horses recorded in training at licensed premises in Australia. These findings, together with more than half of the potentially exiting horses recorded as exiting racing indicates that further improvements are needed, to ensure timely reporting of horses entering and exiting training stables.

The results presented in the current study are a description of barriers to entering training, of the reasons why horses left racing and training, and outcomes for horses upon leaving the TB industry. Documenting and understanding the reasons why horses do not transition from the stud farm to the racetrack is an important first step in developing strategies to mitigate losses, improve productivity in the TB breeding industry and inform discussions concerning the welfare outcomes for these horses. The major barrier to entering training in the surveyed population was that the horse had died, with the majority of these deaths occurring prior to the commencement of training. Similarly, understanding the reasons that racehorses leave training and racing, and reporting the post-racing outcomes for racehorses establishes a benchmark, from which the efficacy of any new strategies can be evaluated. The most important finding of the current study was that approximately three quarters of trained horses were re-homed upon leaving the racing industry. Also, it is important to note was that the majority of TB horses that did not return to the breeding industry were engaged in some form of ridden equestrian activity after their racing career was over. Previous studies that investigated and described these parameters have used the term ‘wastage’ to describe the proportion of the population that did not race, or the proportion of the racing population that left racing [15, 16, 25–27]. More recently, however, the term ‘wastage’ has been considered inappropriate for use in epidemiological studies that do not assess the economic aspects of production losses [28]. When research findings are translated into more mainstream media coverage, the term ‘wastage’ implies a disregard by the TB industry for horses in its care [6, 7, 29–32]. The current study identified that more than 70% of horses that retired or were re-homed in the survey population, were either undertaking some ridden equestrian endeavour or had returned to the stud farm as breeding stock. The large proportion of horses engaged in productive post-race career outcomes is therefore not consistent with a descriptor of ‘wastage’. Benchmarking studies such as this are critical to monitoring current horse welfare standards in the TB industry, and enabling strategies intended to improve welfare to be audited and evaluated in the future.

The Australian Rules of Racing require that horses not start racing prior to 1 October of the two-year-old racing season and cease racing by 31 July of the 12-year-old racing season. The current study identified that a large proportion of horses leave racing around five-years of age. This means that horses are using a relatively small proportion of a potential 11-years of racing. Relatively large numbers of horses retiring for predominately voluntary reasons at five-years of age, suggests that industry-level, rather than individual horse-level biological factors, are the predominant influences on racing career duration. Quantifying the relative contributions of industry-level versus individual horse-level effects, on the decision to cease racing are essential to understand factors contributing to the sustainability of the racing industry as a whole.

There are a number of important limitations in the current study. The selection of the survey participants in this study was based on the enrolment of a specific horse in the study. This approach was similar to that used by Bourke (1995) and Bailey (1999) where individual horses were enrolled in the study from yearling sale cohorts [15, 16]. While participants were assured that their responses would be de-identified, linking of responses to a specific horse may have influenced the responses of some survey participants. The non-response rate for the untrained horses was exacerbated by the large proportion of participants enrolled in the survey, with incorrect, or email-only contact details, which may have introduced selection bias. The enrolment of study participants was restricted to registered breeders and licensed trainers. This may have introduced recall bias if there was a prolonged period since the horse was in their care or if the horse had only been in their care for a short period. The exclusion of horses that were still actively racing in the six months prior to the start of the follow up period, may also have introduced some selection bias for the trained horses, leading to an underestimation of the horses that were participating in the racing industry. The extended time between the date of the horse’s outcome and administration of the survey may have introduced recall bias, though our probabilistic sensitivity analyses show that a substantial proportion of responses would need to have been misclassified to result in a meaningful change in the proportion of horses classified as deceased.

## Conclusions

The most significant barrier to horses transitioning from the stud farm to entering training was death, with half of those deaths occurring in the first year of life. The key driver of exit for trained horses was voluntary retirement, due to poor performance or owner request, with the majority of horses reported as subsequently pursuing ridden activities after retirement. The relatively large number of horses that retired for voluntary reasons from the industry at five-years of age, suggests that industry-level and horse performance influences on racing career duration, play a bigger role in driving retirement than previously thought. Data from this study will provide an important baseline, which will allow any subsequent changes in industry regulation to be audited and evaluated.

## Supporting information

S1 File Survey questions. (PDF)

S2 File Hyperlink to online example survey. (PDF)

S3 File Dataset. (CSV)

## References

[1] Anon. Size and Scope of the Victorian Thoroughbred Racing Industry. https://www.racingvictoria.com.au/about-us/-/media/9effc391608a40f68126ae61be08768e.ashx: IER, 2018.

[2] Anon. Racing Australia Size and Scope Study. https://ier-study.racingaustralia.horse: IER, 2017.

[3] Anon. Commercial horse racing position statement sentient.org.au [accessed 30/3/17]. Available from: http://www.sentient.org.au/commercial-horse-racing-position-stateme.

[4] Anon. Animal welfare in horse racing 2019 [accessed 1/7/2020]. Available from: https://www.rspca.org.au/take-action/animal-welfare-in-horse-racing.

[5] Anon. Horse Racing Animals Australia2020 [accessed 1/7/2020]. Available from: https://www.animalsaustralia.org/issues/horse_racing.php.

[6] Fox Koob S. How is horse racing cruel? The Sydney Morning Herald. 18/10/2019; Sect. Racing Integrity.

[7] Wahlquist C. Five ways to make horse racing more humane right now. The Guardian. 4/11/2019.

[8] Anon. Wastage [accessed 30/3/17]. Available from: https://www.horseracingkills.com/campaigns/wastage/.

[9] Gunningham N, Kagan R, Thornton D. Social License and Environmental Protection: Why Businesses Go Beyond Compliance. Law & Social Inquiry. 2004;29(2):307–41.

[10] Anon. Australian Racing Board Fact Book 07/08. Annual Report. Publishing Services RISA.com: Australian Racing Board, 2008.

[11] Anon. Racing Australia Fact Book: A Guide to the Thoroughbred Industry in Australia 2017/2018. Publishing Services, RISA.com: Racing Australia, 2018.

[12] Anon. What are the animal welfare issues with Thoroughbred horse racing? RSPCA Knowledgebase: RSPCA; 2019 [updated 24 December 2019; accessed 25/02/2020]. Available from: https://kb.rspca.org.au/knowledge-base/what-are-the-animal-welfare-issues-with-thoroughbred-horse-racing/.

[13] Anon. What is horse 'wastage' in the racehorse industry? RSPCA Knowledgebase: RSPCA; 2016 [updated 22 March 2016; accessed 30/3/17]. Available from: http://kb.rspca.org.au/What-is-horse-wastage-in-the-racehorse-industry_235.html.

[14] Wilsher S, Allen WR, Wood JL. Factors associated with failure of thoroughbred horses to train and race. Equine Veterinary Journal. 2006;38(2):113–8. 10.2746/042516406776563305 . 16536379

[15] Bourke J, editor Wastage in Thoroughbreds. Annual Seminar of the Equine Branch, New Zealand Veterinary Association; 1995 1995; New Zealand. Proceedings of the Annual Seminar of the Equine Branch of New Zealand Veterinary Association: New Zealand Veterinary Association; 1995.

[16] Bailey CJ, Reid SW, Hodgson DR, Rose RJ. Factors associated with time until first race and career duration for Thoroughbred racehorses. American Journal of Veterinary Research. 1999;60(10):1196–200. . 10791929

[17] Flash ML, Crabb HK, Hitchens PL, Firestone SM, M.A. S, Gilkerson JR. Unpublished Results. 2020.

[18] Thomson PC, Hayek AR, Jones B, Evans DL, McGreevy PD. Number, causes and destinations of horses leaving the Australian Thoroughbred and Standardbred racing industries. Australian Veterinary Journal. 2014;92(8):303–11. 10.1111/avj.12204 . 24954530

[19] Perkins NR, Reid SWJ, Morris RS. Profiling the New Zealand Thoroughbred racing industry. 2. Conditions interfering with training and racing. New Zealand Veterinary Journal. 2005;53(1):69–76. 10.1080/00480169.2005.36471 15731837

[20] Reed SK, Vander Ley BB, Bell RP, Wilson DA, Wilborn E, Keegan KG. Survey on Thoroughbred use, health and owner satisfaction following retirement from racing. Equine Veterinary Education. 2019;n/a(n/a). 10.1111/eve.13185

[21] Australian Rules of Racing, (2019).

[22] Dohoo I, Matrin W, Stryhn H. Methods in Epidemological Research. McPike SM, editor. Charlottetown, Prine Edwards Island: VER Inc; 2012 2012. 890 p.

[23] Lash TL, Fox MP, Fink AK. Applying Quantitative Bias Analysis to Epidemiologic Data. New York: Springer 2009.

[24] Flash ML, Wong ASM, Stevenson MA, Gilkerson JR. Barriers to entering race training before 4 years of age for Thoroughbred horses born in the 2014 Australian foal crop. PlosOne. 2020; (accepted). 10.1371/journal.pone.0237003 32756576 PMC7406052

[25] Jeffcott LB, Rossdale PD, Freestone J, Frank CJ, Towers-Clark PF. An assessment of wastage in Thoroughbred racing from conception to 4 years of age. Equine Veterinary Journal. 1982;14(3):185–98. 10.1111/j.2042-3306.1982.tb02389.x 7106081

[26] More SJ. A longitudinal study of racing Thoroughbreds: performance during the first years of racing. Australian Veterinary Journal. 1999;77(2):105–12. 10.1111/j.1751-0813.1999.tb11678.x 10078358

[27] Perkins NR, Reid SWJ, Morris RS. Profiling the New Zealand Thoroughbred racing industry. 1. Training, racing and general health patterns. New Zealand Veterinary Journal. 2005;53(1):59–68. 10.1080/00480169.2005.36470 15731836

[28] Parkin TDH, Rossdale PD. Epidemiology of equine performance wastage: importance of analysing facts and implementing their message in management. Equine Veterinary Journal. 2006;38(2):98–100. 10.2746/042516406776563279 16536374

[29] Gemmell N. It’s un-Australian, I know, but …. The Australian. 4/11/17.

[30] Lynch M. Racing must confront its ‘wastage’ problem quickly. The Sydney Morning Herald. 18/10/19.

[31] Manning J. This is the racing industry’s biggest, darkest secret. The Sydney Morning Herald. 15/11/12.

[32] Tigerlily DJ. I’m boycotting horseracing because it’s lethal. The Sydney Morning Herald. 21/10/19.

