# Supplementary material for: Descriptive analysis of Thoroughbred horses born in Victoria, Australia, in 2010; barriers to entering training and outcomes on exiting training and racing
Source: PLoS One. 2020 Oct 28;15(10):e0241273. doi: 10.1371/journal.pone.0241273 (PMC7592779; doi:10.1371/journal.pone.0241273)
Supplement: S2 File — (PDF) [file pone.0241273.s002.pdf]

S2 File: Hyperlink to online example survey

https://www.surveymonkey.com/r/TK2MS6Q?&n=Fast%20As&s=Quick%20Boots&d=Lightning%20McQueen&sx=Male&db=1/5/2016&cr=Pink%20Striped
